# Minimally Invasive Plasma Device Management of Multiple Benign Skin Cancers Associated with Rare Genodermatoses—Case Series and Review of the Therapeutic Methods

**DOI:** 10.3390/jcm13154377

**Published:** 2024-07-26

**Authors:** Anna Płatkowska, Monika Słowińska, Joanna Zalewska, Zbigniew Swacha, Anna Szumera-Ciećkiewicz, Michał Wągrodzki, Janusz Patera, Katarzyna Łapieńska-Rey, Małgorzata Lorent, Iwona Ługowska, Piotr Rutkowski, Witold Owczarek

**Affiliations:** 1 Department of Dermatology, Military Institute of Medicine—National Research Institute, Central Clinical Hospital Ministry of Defense, Szaserow 128, 04-141 Warsaw, Poland; aplatkowska@wim.mil.pl (A.P.); jzalewska@wim.mil.pl (J.Z.); zswacha@wim.mil.pl (Z.S.); wowczarek@wim.mil.pl (W.O.); 2 Anclara Health & Aesthetic lek. Anna Płatkowska, Anclara Ltd., Puławska 136/62, 02-511 Warsaw, Poland; 3 Department of Pathology, Maria Sklodowska-Curie National Research Institute of Oncology, Roentgena 5, 02-781 Warsaw, Poland; anna.szumera-cieckiewicz@nio.gov.pl (A.S.-C.); michal.wagrodzki@nio.gov.pl (M.W.); 4 Biobank, Maria Sklodowska-Curie National Research Institute of Oncology, Roentgena 5, 00-001 Warsaw, Poland; 5 Department of Pathology, Military Institute of Medicine—National Research Institute, Central Clinical Hospital Ministry of Defense, Szaserow 128, 04-141 Warsaw, Poland; jpatera@wim.mil.pl (J.P.); klapienska-rey@wim.mil.pl (K.Ł.-R.); 6 Department of Pathology, National Research Institute of Tuberculosis and Lung Diseases, Płocka 26, 01-138 Warsaw, Poland; m.lorent@igichp.edu.pl; 7 Department of Soft Tissue, Bone Sarcoma and Melanoma, Maria Sklodowska-Curie National Research Institute of Oncology, Roentgena 5, 00-001 Warsaw, Poland; iwona.lugowska@nio.gov.pl (I.Ł.); piotr.rutkowski@nio.gov.pl (P.R.); 8 Early Phase Clinical Trials Unit and Department of Soft Tissue/Bone Sarcoma and Melanoma, Maria Sklodowska-Curie National Research Institute of Oncology, Roentgena 5, 02-781 Warsaw, Poland

**Keywords:** Birt–Hogg–Dubé syndrome, Cowden syndrome, Muir–Torre syndrome, nevus sebaceous syndrome, neurofibromatosis, Brooke–Spiegler syndrome, treatment, plasma device, genodermatoses, adnexal tumours, non-melanoma skin cancers

## Abstract

**Background**: Non-melanocytic benign skin tumours encompass a diverse group of lesions, classified based on their cellular origin, such as epidermal, vascular, fibrous, neural, muscle, and adnexal tumours. Though they often reveal solitary lesions, multiple skin tumours focus on genodermatoses. Each syndrome exhibits distinct clinical characteristics and potential complications, including cutaneous and extra-cutaneous malignancies, some of which are potentially life-threatening. Diagnosing genetic syndromes is complex and requires numerous histopathological and immunohistochemistry tests due to similarities between the adnexal tumours and basal cell carcinoma upon pathology. **Methods**: To illustrate the clinical practice, we conducted a retrospective case study that included eleven patients with genodermatoses referred to a tertiary dermatology clinic from September 2018 to April 2024. We have also conducted a research study on available treatment modalities in this setting. **Results**: Five patients with excellent aesthetic results were treated using a recently approved FDA plasma device. After searching SCOPUS and PubMed database records, we assessed 96 original articles to present current knowledge regarding the dermato-surgical approach. **Conclusions**: Multiple skin tumours, especially on the face, may significantly affect patients’ quality of life and have psychological consequences. An appropriate treatment selection tailored to the patient’s needs should be provided. There is no standardised treatment for multiple benign tumours in genodermatoses, and selected methods with varying efficacy are employed. We presented the utility of a new plasma device in these settings.

## 1. Introduction

Selected rare genodermatoses involve the formation of numerous skin cancers, and solid malignant neoplasms of internal organs during a lifespan [1,2,3]. They require profound diagnostics, gathering a detailed family history, a multidisciplinary approach, close oncological surveillance, and long monitoring, but still, the local treatment is challenging [1,2,3,4]. Benign skin tumours encompass a diverse group of lesions, classified based on their cellular origin, such as epidermal, melanocytic, vascular, fibrous, neural, muscle, and adnexal tumours [1,2,3,4]. Each category exhibits distinct clinical characteristics and potential complications. From the perspective of patient management, the differential diagnosis of the adnexal skin neoplasms occurs to be especially difficult, but crucial in terms of selecting the appropriate dermo-surgical treatment of numerous benign or malignant lesions [4].

### 1.1. Selected Genodermatoses—Diagnostic Criteria and Characteristics

This article focuses on rare genodermatoses manifested by multiple benign skin cancers, which are indicative of profound diagnostics of mostly cutaneous and internal malignancies. It is also essential to be aware that patients suffering from those inherited disorders manifest sometimes severe comorbidities [1,2,3]. Unfortunately, most patients, and their relatives, remain undiagnosed for years despite their seeking for a resolution of cutaneous problems. Therefore, awareness of skin symptoms, which might be overlooked in young adults, when the skin tumours are small and more discrete, is essential for early diagnosis, close monitoring, and treatment, as all influence their outcome and prognosis [1,2,3]. The diagnostic criteria of presented genetic syndromes are presented in Table 1.

Genetic and clinical characteristics of presented genodermatoses in the context of cutaneous and extra-cutaneous malignancies are summarised in Table 2.

### 1.2. Benign Skin Tumours—Histologic and Dermoscopic Diagnostics

The analysis of the spectrum of benign cutaneous tumours related to the presented genodermatoses reveals predominance of their adnexal origin, except for neurofibromatosis type 1, which is related to abnormal differentiation of the neural tissue. Cutaneous adnexal neoplasms are a heterogeneous group of skin tumours of follicular, sebaceous, apocrine, or eccrine origin, which give rise to benign or malignant entities [4]. The diagnosis is challenging due to unspecific clinical presentation, and, therefore, the histopathological examination remains enhanced by the immunohistochemistry staining currently used (Table 3) [4]. The differential diagnosis comprises the description of closely related benign variants (such as trichoblastoma and trichoepithelioma), the co-occurring malignant transformation of one or multiple adnexal components, called hybrid/composite tumours (as in epidermal or sebaceous nevus syndrome), and the appearance of basal or squamous cell carcinomas arising on their basis [4,19,20,21,22]. Therefore, additional application of dermoscopy might be helpful to rule out common skin cancers (BCC, SCC, melanoma). Based on the presence of the ulceration, polymorphism of vessels, or whitish structures, in larger size lesions, the suspicion of the malignant adnexal tumour should be made before the local dermato-surgical procedures with ablative devices (Table 3) [23,24].

Adnexal tumours, both benign and malignant, represent only 1 to 2% of all diagnosed skin cancers [4,40]. The typical location is the head and trunk. Malignant adnexal tumours are reported as non-symptomatic, slow-growing nodules, locally aggressive with the potential for local and distant metastasis resulting in poor outcomes [4]. They develop from a pre-existing benign tumour or arise de novo, in middle-aged (with the peak in the eighth decade) patients, though there are also reports of paediatric cases [4,40,41,42]. In case of the adnexal tumours arising on a genodermatosis background, the onset starts in childhood or above the age of 20 years, respective to its characteristics [1,2,4,5].

### 1.3. Screening and Management of Selected Genodermatoses

The literature underlines the necessity of multidisciplinary care of patients with genodermatoses [1,2,3,43,44,45]. Guidelines describing the screening and management are available only for selected disorders, such as for Cowden syndrome by NCCN [43]. As early detection is crucial, once the diagnosis is made, screening and genetic counselling should follow, and management should be determined on a case-by-case basis [1,2,3,5]. Generally, the differences consist of the age ranges in which the annually repeated examinations of skin and internal organs should start.

The NCCN recommendations for cancer surveillance in Cowden syndrome are annually revised and include the following: for children (<18 years): yearly thyroid ultrasound and skin check with physical examination; for adults: yearly thyroid ultrasound and dermatologic evaluation; women beginning at the age of 30 years: monthly breast self-examination, annual breast screening (at minimum mammogram; MRI may also be incorporated) and transvaginal ultrasound or endometrial biopsy [44]. For men and women: colonoscopy beginning between the ages of 35 and 40 with frequency depending on the degree of polyposis identified; biennial (every 2 years) renal imaging (CT or MRI preferred) beginning at the age of 40 years [44]. An alternative schedule of the screening examination was provided by Tan MH et al. based on analyses and extensive clinical experience in a prospective series of patients [44]. For advanced mucocutaneous lesions, promising results have been displayed by the use of rapamycin [45].

Based on the literature, in the case of Muir–Torre syndrome, annual (or more frequent if necessary based on history) skin examinations should be performed [8]. Additional screening is recommended based on the risk for visceral malignancy and includes annual laboratory tests beginning at 35 years of age [8]. Annual colonoscopy starts at 20 years of age or 2 to 5 years younger than the youngest age of colorectal cancer diagnosis in the family. Annual breast and pelvic examinations in women are recommended to screen for breast and ovarian cancer, and annual endometrial biopsy and transvaginal ultrasonography should also be initiated at 30 years of age, especially for women with proven genetic mutations. Annual prostate and testicular examinations for men are also advised [8].

The management of NFt1 involves annual MRI for high-risk children beginning between the ages of 10 and 12 years, and in all patients who meet diagnostic criteria for the disease [46,47,48,49]. Treatment may involve debulking of larger tumours, though the plexiform neurofibromas are difficult to resect due to intimate intertwining with neurovasculature. The main concern during monitoring is the diagnostics of malignant peripheral nerve sheath tumours, breast cancer, and psychiatric and neurologic diagnoses [1,2,3,5,46,47,48,49]. Therefore, patients and family members should be educated on these conditions and their symptoms. Current research is focused on pharmacologic treatments that inhibit the MEK pathway, and early results with selumetinib and trametinib are promising [49].

In case of mosaic syndromes, the underlying mutation occurs in an oncogene, resulting in an increased risk of malignancies [50]. Phacomatosis pigmentokeratotica is a sporadic acquired mosaic disorder characterised by the co-occurrence of a widespread nevus sebaceous, along with a speckled lentiginous nevus, both sharing the same postzygotic activating mutations in HRAS [51].

Therefore, annual skin examination especially with use of a dermoscope is important in aiming to early reveal the spitzoid melanomas within the speckled lentiginous nevus [51]. Other tumours associated with RAS overactivity, such as rhabdomyosarcoma, have also occurred in these patients [50]. Although guidelines for screening do not presently exist, it is important to consider that these “mosaic RASopathies” may carry an increased risk of internal malignancy. The risk of internal malignancy depends on the extent of somatic mosaicism in both cell number and cell types/organs effected, and, as of now, there is no clear way to predict that based on the cutaneous lesions [50].

For patients diagnosed with Birt–Hogg–Dubé syndrome (BHDS), in about 25% of cases, severe complications manifest that are related to renal cancer and/or pulmonary spontaneous pneumothorax from the rupture of pulmonary cysts [1,2,3,5]. Therefore, the patients are advised not to smoke and to undergo annual MRIs or CT scans [3]. Potential associations with the risk of colon or breast cancer have been recently reported [5]. There is no clear evidence regarding imaging renal follow-up: the latter can be very different, ranging from every 3–6 months to every 2–3 years, depending on the size of lesions [3]. Surveillance could be started at 30–35 years of age and/or 10 years before the earlier age of onset of an RCC in a given family.

Brooke–Spiegler syndrome (BSS) patients need regular surveillance to detect potential malignant salivary gland tumours or basal cell carcinoma (BCC), and minimise disfigurement caused by the growth of multiple skin tumours within the head [1,2,3,5]. The exact risk remains unknown, as it is impossible to detect the putative mutation due to a variety of alternative mechanisms and/or incomplete sensitivity of the genetic technique [45,51,52].

During the life-time course of genodermatoses, the cutaneous tumours usually appear from childhood to the third decade, or start above 20 years of age, and tend to gradually grow in size and number throughout life [1,2,3,5]. Moreover, pre-existing benign adnexal tumours hide a malignant potential, which may reveal within single or multiple skin lesions [1,2,3,4]. It was confirmed that skin diseases, and particularly the genetic syndromes, may significantly affect the quality of life of patients, and have psychological consequences such as depression and anxiety [53]. Additionally, patients with numerous visible tumours may experience various forms of stigmatisation, such as social disapproval, discrediting, or devaluation. Therefore, an individualised approach should also be underlined in managing this setting. In this aspect, the achievement of satisfactory therapeutic and aesthetic results is particularly important within the face, and might be very important for our patients.

This research paper explores the intricacies of diagnosing and managing of rare non-malignant skin tumours, focusing on contemporary dermo-surgical techniques. The presented case series provides insight into clinical practice with patients suffering from genodermatoses with related uncontrolled development of benign cutaneous neoplasms.

## 2. Patients and Methods

### 2.1. Case Study

The retrospective case study was based on medical documentation (epidemiological, anamnestic, familial, clinical, videodermoscopic, histopathologic, and ultrasound/computer tomography results) of consecutive patients who were diagnosed with genodermatosis associated with cancers between September 2018 and April 2024 in the tertiary dermatologic clinic. The clinical and dermoscopic records were received from Fotofinder Medicam HD 1000 (FotoFinder Systems GmbH, Bad Birnbach, Germany). The diagnosis was based on the diagnostic criteria of selected genetic syndromes, and histopathologic and dermoscopic structures of associated benign skin tumours presented above in Table 1, Table 2 and Table 3.

The Plasma IQ^®^ (Berger & Kraft Medical, Ltd., Warsaw, Poland) was used to perform dermatological treatment of benign skin tumours upon patient request. The procedure was performed under local anaesthesia with an anaesthetic cream (Emla^®^) or subcutaneous injection of 1% solution of lidocaine. After the procedure, the patients were asked to describe the severity of pain according to the NRS scale [54].

### 2.2. Review of Therapeutic Methods Based on Research Study

A research study on selected genodermatoses was conducted on 15 May 2024 by AP. The search included the terms “genodermatosis and treatment”, “Birth-Hogg-Dube syndrome and treatment”, “Muire-Torre and treatment”, “Phacomatosis pigmentovascularis and treatment”, “Cowden syndrome and treatment”, “Neurofibromatosis type I and treatment”, “Brooke-Spiegler syndrome and treatment”, “benign adnexal tumours and treatment”, “benign skin tumour and treatment”, “cylindroma and treatment”, “fibrofolliculoma and treatment”, “sebaceous nevus and treatment”, “epidermal nevus and treatment”, “spiradenoma and treatment”, “syringocystadenoma papilliferum and treatment”, “trichoblastoma and treatment”, “trichilemmoma and treatment”, “trichoepithelioma and treatment”, “neurofibroma and treatment”, “laser and benign skin tumour”, “radiofrequency and benign skin tumour”, “electrodesiccation and benign skin tumour”, “plasma device and benign skin tumour”, and “energy based device and benign skin tumour” in articles written in English in the SCOPUS and PubMed databases.

**Figure 1:**
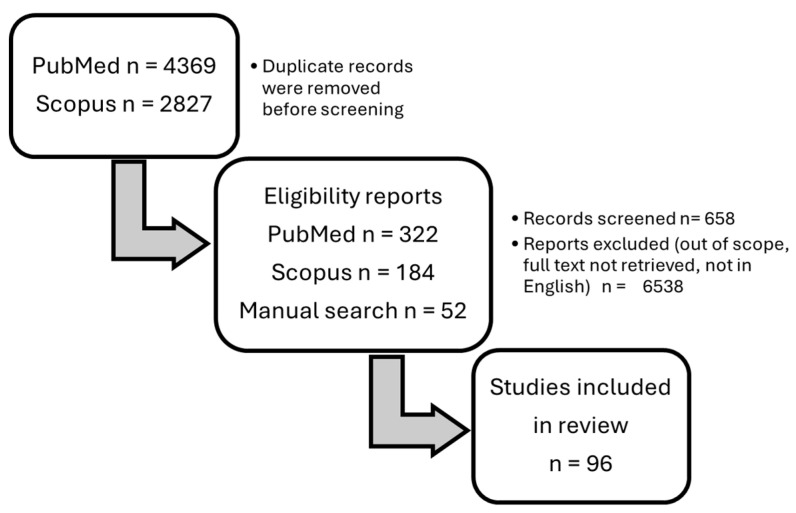
Flowchart on the selection and evaluation of scientific articles.

In total, 558 potentially eligible articles were detected. Additional relevant publications were obtained by reviewing the references from the chosen articles. After the removal of duplicates and irrelevant articles based on the titles, abstracts, and full-text articles, 658 records were screened, and 98 original articles were included in this review (Figure 1).

## 3. Results

### 3.1. Case Study

Eleven patients referred to the dermatologic clinic with the intention of diagnosing and cure skin tumours and who were diagnosed of genodermatosis associated with cancers were included in this case study. All patients presented selected types of multiple benign skin tumours—fibrofolliculomas, trichoepitheliomas, trichoblastomas, trichilemmomas, sebaceous adenomas, sebaceomas, syringocystadenomas papilliferum, neurofibromas, cylindromas, spiradenomas, and nevus sebaceous syndrome. In three patients (with CS and MTS, and PPK), additional revision of previous pathological reports was necessary to obtain the final diagnosis. Among the first-step relatives in eight patients (BHDS—two patients, CS—one patient, BSS—five patients), the genetic syndromes were also confirmed. In three cases, a prior diagnosis of cancer were obtained before referral to our clinic. The patient with CS was diagnosed with breast and endometrial cancers. The patient with MTS was diagnosed with sebaceous cancer of the lower limb. One of the skin lesions in the case of PPK was diagnosed as syringocystadenocarcinoma papilliferum. No cases of BCC were revealed among patients with BSS or PPK. All patients remain under surveillance of the dedicated specialists.

Five patients underwent removal of the multiple benign skin tumours with the use of the Plasma IQ^®^ (Berger & Kraft Medical, Ltd., Warsaw, Poland) under local anaesthesia, with acceptable pain control (NRS pain scale of 4–6/10), short postsurgical recovery (5–10 days), and good aesthetic effect. Patients with BSS underwent surgical excisions or electrodesiccation of selected tumours, as a consequence of their size and vascularity. All lesions removed in patients with BSS and PPK were histopathologically revised to exclude the malignant transformation of skin tumours.

#### 3.1.1. Birt–Hogg–Dubé Syndrome (BHDS)

**Figure 2:**
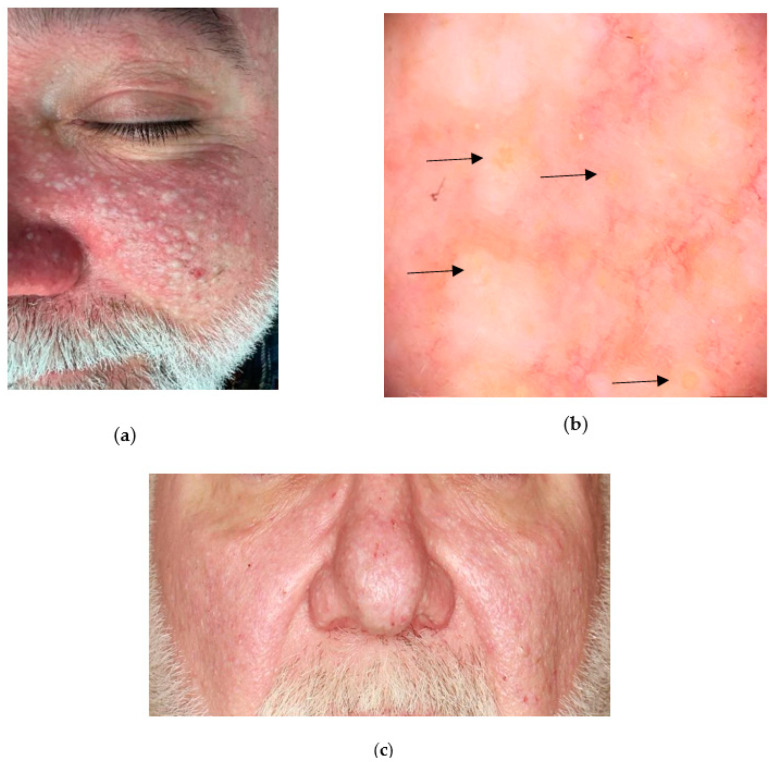
The case of the Birt–Hogg–Dubé syndrome: (**a**) clinical presentation of multiple fibrofolliculomas on the face; (**b**) videodermoscopy (Fotofinder Medicam HD 1000 Systems GmbH, Bad Birnbach, Germany) revealed small nodules composed of fibrosis (white opalescent colour) and acanthosis of the epithelium (yellow colour) at the level of hair openings or the infundibulum; at the top of some nodules a keratotic plug in the hair follicle opening was visible (arrow); (**c**) final aesthetic result of the procedure with Plasma IQ^®^ (Berger & Kraft Medical, Ltd., Warsaw, Poland).

A 46-year-old male patient was referred to our clinic due to numerous, cohesive flesh- coloured and pearly nodules up to 5 mm in diameter on the skin of the head and neck (Figure 2a). The first symptoms appeared on the face at the age of 26. Since then a gradual increase in the number of lesions proceeded; therefore, the patient looked for a diagnosis and therapy. Primary, he was diagnosed with post-acne scarring, and treated with a low dose of isotretinoin (20 mg/day) for a few months without result. The patient had no other complaints or accompanying symptoms at that time. The diagnostics started with a dermoscopic examination and punch biopsy of the skin lesion. Videodermoscopy suggested fibrosis of hair follicle openings or the infundibulum (Figure 2b). Additionally, no atypical blood vessels were detected, which excluded diagnosis of trichoblastoma or trichoepithelioma. The histopathological examination revealed a diagnosis of fibrofolliculoma. As the multiple lesions located within the face, ears, and head caused an aesthetic impairment for the patient, we removed them with the plasma device under local anaesthesia. The patient’s tolerance of the procedure was good, with an NRS pain scale of 4/10. Postsurgical recovery lasted up to 5 days, and the aesthetic effect was excellent (Figure 2c). Within one year of follow-up, no recurrences of removed lesions were observed. Due to suspicion of BHDS, which predisposes people to kidney tumours and lung cyst development, the patient and his first-step relatives underwent profound diagnostics. Abdominal ultrasound revealed a liver cyst on the border of the right and left lobes of dimension 20 × 23 mm. In addition, the left kidney contained a cortical cyst with a diameter of 16 mm in the upper pole. Contrast-enhanced chest computed tomography revealed cysts in both lungs, including the largest at the base of the fifth segment of the right lung (approximately 13 × 24 mm). The patient was referred to genetic, nephrological, and pulmonological counselling for monitoring, and follow-up of the detected lesion.

We managed to also reveal BHDS in the case of the patient’s mother, based on diagnostic criteria such as numerous nodules in the area of the face, neck, and chest of the fibrofolliculoma type, and lung cysts with a propensity for pneumothorax. At the moment of submission of this article, none of the patients were diagnosed with kidney cancer.

#### 3.1.2. Cowden Syndrome (CS)

**Figure 3:**
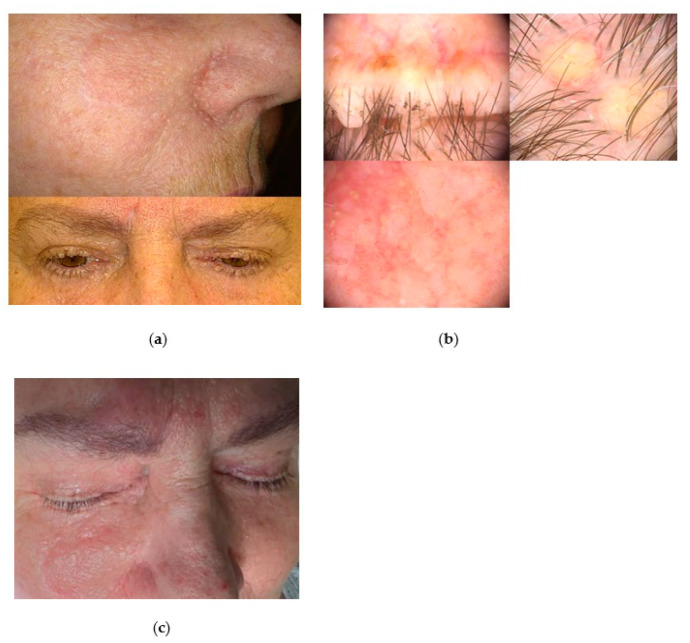
The case of Cowden syndrome. (**a**) Clinical presentation of multiple tumours originated from the hair follicles (trichoblastomas, trichoepitheliomas) on the face and the eyelids; (**b**) videodermoscopy (Fotofinder Medicam HD 1000 Systems GmbH, Bad Birnbach, Germany) revealed small pearl and yellow coloured nodules at the rim of eyelids and skin-coloured tiny nodules on the nose ala; (**c**) final aesthetic result of the procedure with Plasma IQ^®^ (Berger & Kraft Medical Ltd., Warsaw, Poland).

A 63-year-old female patient was referred to our clinic due to multiple pearl and yellow-coloured, small (a few millimetres in size) nodular lesions at the rim of the eyelids and scalp (Figure 3a). Skin-coloured tiny nodules were also scattered on the nose, cheeks, and the forehead.

During the 18 years before hospitalisation, the patient underwent multiple surgical excisions on the face and scalp area, with histopathological diagnoses of BCC, trichoepithelioma, trichoblastoma, and trichilemmoma. As one of the BCC was potentially locally advanced, the patient was considered as candidate for vismodegib therapy. The most disturbing for the patient were the lesions growing at the edge of the eyelids, which, upon videodermoscopic examination, might correlate with the sebaceous adenomas (Figure 3b) due to yellow colour and their location. The clinical manifestation of those lesions was suggestive for MTS.

The revision of the pathological specimens was performed, and the previous BCC diagnoses were changed to trichoblastomas. The medical history revealed mastectomy of the left breast with subsequent chemotherapy due to breast cancer, and hysterectomy due to endometrial cancer. The patient was also diagnosed with rheumatoid arthritis, depression, and cervical and lumbar discopathy. The family history revealed the patient’s son, who presented with similar skin lesions and was diagnosed with a brain tumour (Lhermitte–Duclos disease was suspected) and mental retardation. As the skin tumours located on the edge of the eyelids caused considerable discomfort for the patient and limited her field of vision, they were removed with a plasma device under local anaesthesia. The patient’s tolerance of the procedure was good, with an NRS pain scale of 5/10. Postsurgical recovery lasted up to 7 days, and the aesthetic effect was excellent (Figure 3c). Within the three years of follow-up, no recurrences of removed lesions were observed.

This case indicates how important it was to choose the appropriate therapeutic method, which is precise, fast, and safe. In the next step, the remaining skin tumours on the face and scalp were removed upon the patient’s request.

The patient was referred for genetic and oncological counselling, but no additional cancers were revealed at the moment of article submission.

#### 3.1.3. Phakomatosis Pigmentokeratotica (PPK), Epidermal Nevus Syndrome/Linear Nevus Sebaceous Syndrome

**Figure 4:**
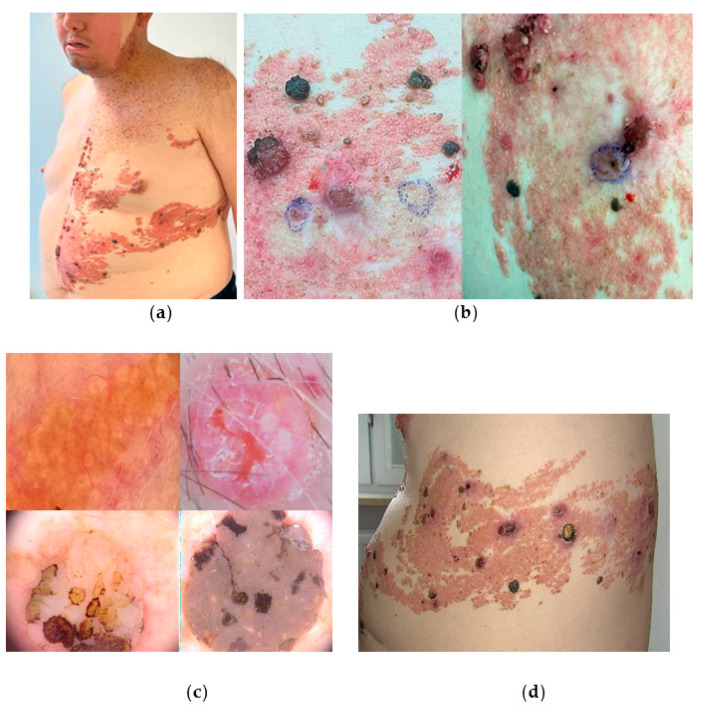
The case of Phakomatosis pigmentokeratotica in lateralisation pattern. (**a**) Clinical presentation of the left side of body—the segmental nevus spilus on the chest, and the segmental sebaceous nevus on the trunk; (**b**) the surface of the sebaceous nevus was covered by numerous, ulcerated tumours, bleeding upon touch—vascular malformations and syringocystadenoma papilliferum; (**c**) the videodermoscopic examination revealed all components: nevus sebaceous—agminated yellow clods surrounded by linear vessels (**upper left**); syringocystadenoma papilliferum—nodular lesion with pinkish and white background, and pearl-like structures at the rim of the centrally located ulceration (**upper right**); and seborrheic keratoses—keratotic plugs on the cerebriform surface (**lower left**) and keratotic and milia-like cysts within structureless brow area (**lower right**); (Fotofinder Medicam HD 1000 Systems GmbH, Bad Birnbach, Germany); (**d**) clinical presentation directly after the plasma device procedure.

A 39-year-old male patient diagnosed with PPK in childhood was referred due to numerous bleeding nodular lesions developed within the segmental sebaceous nevus. Clinically, within the left side of the body, epidermal (sebaceous) nevus syndrome on the trunk, and speckled lentiginous nevus on the head, neck, and chest were present (Figure 4a,b).

Nodular hyperkeratotic lesions were found within the forehead and scalp. Additionally, skull abnormalities were found, manifested by asymmetry and facial deformation. The patient underwent numerous surgical procedures (plastic and reconstructive) on the face and body with the aim of removing the bleeding lesions. In the previous histopathological analysis, one syringocystadenocarcinoma papilliferum in situ within a syringocystadenoma papilliferum lesion was diagnosed. The remaining lesions were benign. Frequent recurrences of bleeding after little traumatisation harmed the patient’s quality of life and daily functioning. Among the remarkable findings reported in his medical history, the patient underwent pericarditis and meningitis in the past, venous thrombosis of the left lower limb with subsequent pulmonary embolism, post-thrombotic syndrome, persistent swelling, and verrucous pyoderma. The patient suffered also from drug-resistant epilepsy. We performed videodermoscopic examination with mapping of all types of secondary skin tumours (Figure 4c), and, subsequently, 14 punch biopsy excisions were carried out of larger tumours with aim of ruling out syringocystadenocarcinoma. The histopathological examination confirmed syringocystadenoma papilliferum in 13 samples, though in 2 of them features of atypia were present, and disturbances in epithelial architecture were described. Smaller diameter lesions, dermoscopically correlating with syringocystadenoma papilliferum, were treated with plasma device under local anaesthesia, with good aesthetic results, and NRS 6/10 (Figure 4d). The patient remains under dermatological surveillance, requiring dermoscopic monitoring and occasional removal of suspicious or troublesome skin lesions.

#### 3.1.4. Muir–Torre Syndrome (MTS)

**Figure 5:**
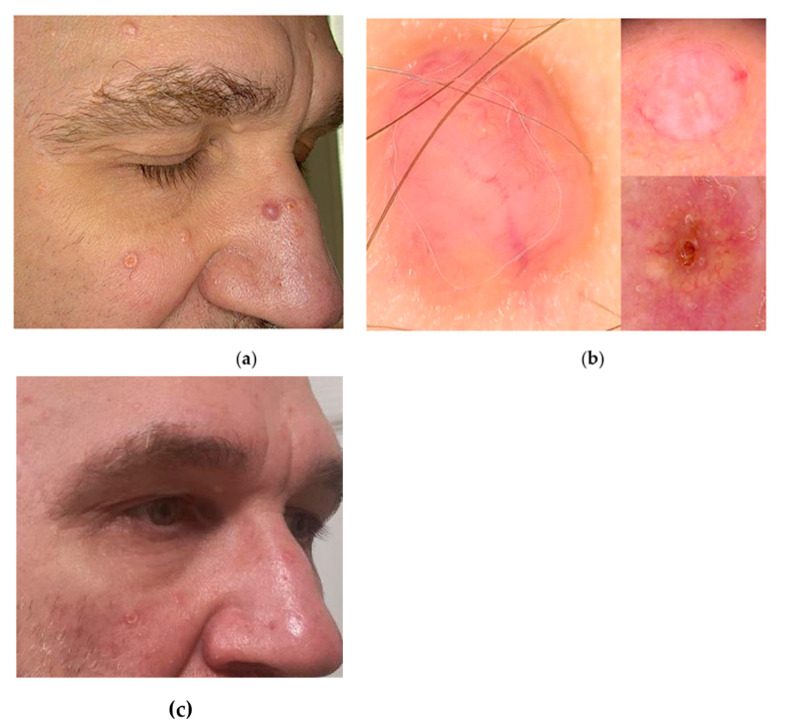
The case of Muir–Torre syndrome: (**a**) clinical presentation of the face with numerous small yellowish nodules with umbilical centres and larger pink nodules with visible telangiectasias on the surface; (**b**) the videodermoscopic examination enabled the differential diagnosis of the lesions—pink nodule with thin and large arborising vessels and yellowish blotches located peripherally, corresponding to larger sebaceous adenoma (on **left**), pink and white in colour nodule with visible withe strands, yellow micro-blotches, and irregular short vessels at the walls of the nodule corresponding to small sebaceous adenoma (**upper right**), yellow clods with crown vessels and central hair follicle opening—corresponding to the sebaceous hyperplasia (on **lower right**) (Fotofinder Medicam HD 1000 Systems GmbH, Bad Birnbach, Germany); (**c**) the aesthetic result two weeks after removal of the sebaceous adenoma of the nose and hyperplasia sebaceous of the forehead with plasma device.

A 54-year-old male patient diagnosed with multiple sebaceous adenomas of the face, trunk, and extremities within the last 8 years, was referred for dermoscopy and reflectance confocal microscopy examination, with the aim of obtaining a differential diagnosis of multiple skin tumours (Figure 5a). In the anamnesis, we discovered the patient underwent right hemicolectomy and adjuvant chemotherapy (Folfox4), due to colon cancer (adenocarcinoma partially mucocellulare G3) with metastasis to mesenteric lymph nodes (pT2N1bM0), at the age of 45, and had a recurrence of the sebaceous adenocarcinoma of the left thigh diagnosed at the age of 53, which were crucial for suspicion of the MTS. The family history revealed four members from the mother’s line diagnosed with colon cancer but without the cutaneous manifestations of the sebaceous neoplasms.

The videodermoscopy enabled us to reveal sebaceous adenomas and sebaceous hyperplasia (Figure 5b). Based on the clinical and videodermoscopic examination, any of the lesions were ulcerated or presented polymorphous vessels, which might be suggestive for sebaceous carcinoma. Due to the recurrence of sebaceous cancer on the left thigh and suspicion of metastases to subcutaneous tissue (of the area of the left chest and axilla, lumbosacral area) detected on PET-CT, the patient was referred to the oncology centre to enrol in a clinical trial. The diagnosis of MTS was confirmed by genetic test. Both the patient and his family were directed for close surveillance according to the NCCN screening schedule in genetic counselling. The patient asked for removal of the sebaceous adenoma of the nose and a few sebaceous hyperplasia of the forehead. The procedure was performed with the plasma device, and its aesthetic result after two weeks follow-up is presented in Figure 5c.

#### 3.1.5. Neurofibromatosis Type 1 (NFt1)

**Figure 6:**
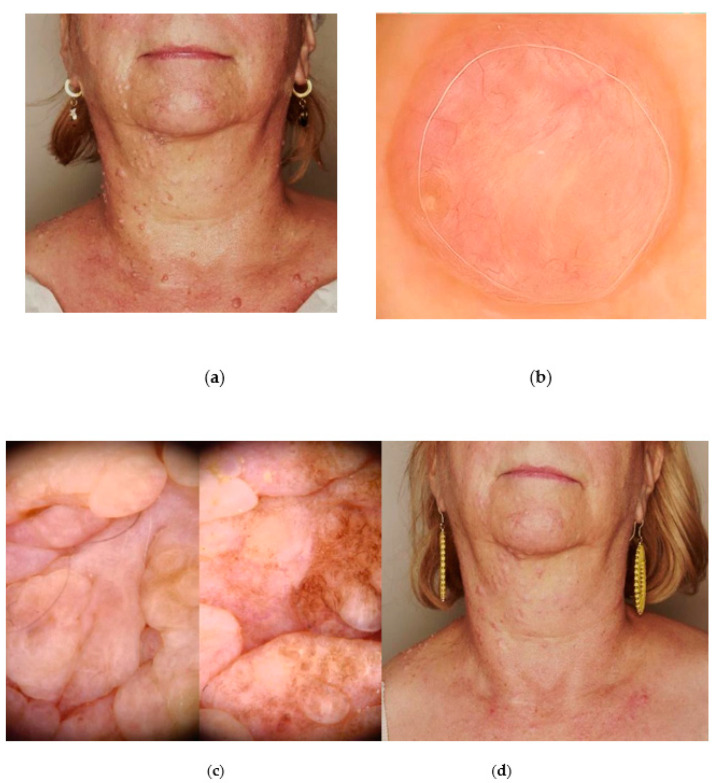
The case of neurofibromatosis type I. (**a**) Clinical presentation reveals multiple pink and skin-coloured soft nodules within the head, neck, and trunk area; (**b**) the videodermoscopy of solitary neurofibroma—pink structureless areas, white strands (scar-like areas), thin linear irregular vessels visible on the surface of nodule; (**c**) the videodermoscopy of plexiform neurofibroma—pink-white structureless areas with fissures corresponding to the flexural surface of the lesion, and peripherally visible brown pigmentation within homogeneous or fingerprint-like structures (Fotofinder Medicam HD 1000 Systems GmbH, Bad Birnbach, Germany); (**d**) the aesthetic result after single procedure with plasma device revealed presence of uneven skin surface, indicating the need for further correction.

A 64-year-old female patient diagnosed with NFt1 since childhood was referred to our clinic with the aim of removing multiple nodules disseminated over the skin surface. The patient considered those lesions as stigmatising her face, neck, and trunk, which harmed her quality of life (Figure 6a). Some neurofibromas were located within the area of café-au-lait spots. Though most of the lesions were solitary, single neurofibromas were plexiform but small in size (Figure 6b,c). The patient had no family history of the disease, as well co-morbidities, including the malignancies, including the spectrum of NFt1. The neurofibromas were removed with the plasma device under local anaesthesia. The patient’s tolerance of the procedure was good, with an NRS pain scale of 4/10. Postsurgical recovery lasted up to 10 days, and the aesthetic effect was very good, although some dome-shaped areas require an additional procedure to achieve a smoother, even surface (Figure 6d). Unfortunately, the removal of solitary neurofibromas is not radical, and results in a high rate of recurrences. The patient was referred to an ophthalmologist, neurologist, and genetic specialist with the aim of early detection of secondary malignancies.

#### 3.1.6. Brooke–Spiegler Syndrome (BSS)

**Figure 7:**
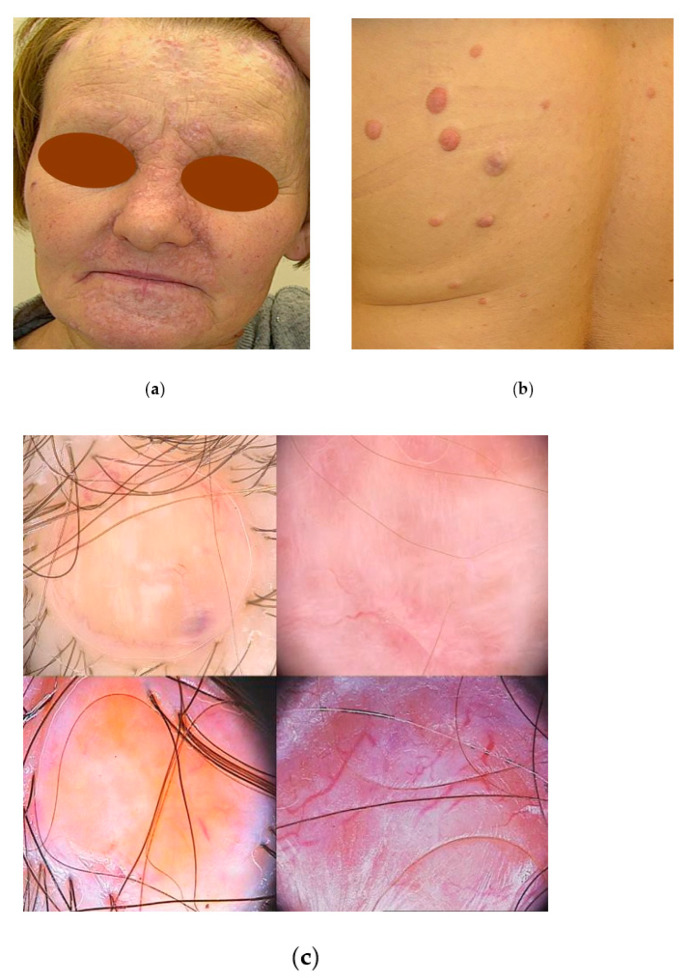
The case of Brooke–Spiegler syndrome: (**a**) clinical presentation—multiple skin-coloured nodules within the area with a high distribution of sebaceous glands on the face; (**b**) clinical presentation—multiple nodules of divert size, pink and skin-coloured, and solid in palpation, located on the trunk; (**c**) videodermoscopy reveals different aspects of cylindromas—pinkish and yellowish structureless areas with blue globule aside of nodule, white blotches and strands on its surface, and short curved or irregular vessels mainly at the rim of nodule (**upper left**), structureless pink and white area with white strands and linear vessels (**upper right**), pink nodule with the centrally yellow structureless area, white strand and peripherally visible short linear vessels (**lower left**), larger nodule, pink and white in colour with large arborising vessels (**lower right**) (Fotofinder Medicam HD 1000 Systems GmbH, Bad Birnbach, Germany).

A 59-year-old female patient was referred for non-invasive examination and treatment of multiple skin-coloured tumours present in the head and trunk area (Figure 7a,b). The videodermoscopy enabled us to reveal of different aspects of cylindromas and helped rule out the presence of basal cell carcinomas (Figure 7c). With the aim of obtaining the final diagnosis and identifying the symptoms of neurofibromas, surgical excisions and electrodissections of lesions were performed. The histopathology confirmed the diagnosis of cylindromas. As numerous relatives of the patient presented similar symptoms, the genetic confirmation of the Brooke–Spiegler syndrome was also performed. Unfortunately, the removal of solitary cylindromas is not radical, and frequently results in recurrences, though in the case of large lesions localised on the scalp, the treatment brings the patient relief of troublesome and seldom ulcerated tumours. Both the patient and her family were taken under close surveillance by a dermatologist, laryngologist, and gastroenterologist to achieve early diagnosis and treatment of potential malignancies.

### 3.2. Review of the Dermatosurgical Treatment Modalities of Benign Skin Tumours in Patients with Presented Genodermatoses

The review of the literature focusing on treatment modalities of benign skin tumours in the setting of genodermatoses resulted in finding limited publications, mostly single-case or case series descriptions. Similarly, results were found when the search was conducted based on the histological type of skin tumours. Table 4 presents the summary of treatment modalities of benign skin tumours, including also selected genodermatoses, based on the performed research search.

Currently, the most commonly applied treatment is surgical excision, removal with non-fractionated ablative carbon dioxide (CO_2_) and erbium:yttrium-aluminium-garnet (Erb:YAG), while neodymium-doped yttrium aluminium garnet (Nd:YAG), potassium-titanyl-phosphate (KTP), or novel picosecond Nd:YAG and dual wave copper vapour lasers are sometimes also used (Table 4). Single reports described the application of radiofrequency (RF) in selected tumours [71,102,103,114]. Interestingly, many publications indicate the use of dermabrasion or cryotherapy in the removal of all investigated cutaneous lesions, including the sebaceous nevus, or cylindromas (some of which are a few centimetres large), despite scarce evidence [1,2,124,125].

According to the S2k guideline regarding the general utility of lasers in the treatment of skin lesions, the removal of the epidermal nevi may be recommended, and sebaceous nevus is recommended, with the use of laser 10,600 nm CO_2_ and 2940 nm Erb:YAG, both traditional ablative and scanned continuous wave [72]. Application of those lasers is also recommended for the removal of neurofibromas and spiradenomas [72]. In the case of spiradenomas, the use of 532 nm KTP laser may also be considered [72]. CO_2_ lasers emit energy, which is selectively absorbed by water within the skin, causing vaporisation, which makes this laser excellent for cutting, coagulation, and ablation of the skin [126]. CO_2_ lasers and Erb:YAG ablative lasers have been reported as effective and fast options for the removal of numerous lesions with few side effects [126,127]. The adverse effects include persistent erythema, hyperpigmentation, hypopigmentation, infections, scarring, acneiform eruptions, milia, and ectropion [72,126,127,128]. To maximise treatment success and minimise complications, detailed preoperative and postoperative care instructions should be reviewed with the patient, including strict sun protection [128].

#### 3.2.1. Birt–Hogg–Dubé Syndrome (BHDS)—Fibrofolliculomas

The literature review revealed a limited number of publications regarding the treatment of fibrofolliculomas in BHDS. Patel et al. presented a case of fibrofolliculomas successfully treated with two sessions of ablative CO_2_ laser therapy, with a 92% reduction in lesion count [55]. Truchuelo et al. reported remission of over 75% of the facial lesions after a single session with ablative CO_2_ laser in two cases, which was maintained after 18 months of follow-up in the first case and four years in the second one [56]. Gambichler et al. reported the utility of Erb:YAG laser in the treatment of lesions that are traditionally difficult to treat (such as ears), albeit with their recurrence after 6 months [60]. Gijezen et al., in a double-blind placebo-controlled randomised split-face trial conducted on 19 patients with BHDS, proved the failure of treatment with the use of 0.1% topical rapamycin for 6 months [61].

#### 3.2.2. Neurofibromatosis Type 1 (NFt1)—Cutaneous Neurofibromas

Management of NFt1 is largely supportive, and cutaneous neurofibromas are frequently removed upon patient request, as they may cause discomfort, itching, and visible disfigurement with constant growth [1,2,46]. Removal of neurofibromas may improve quality of life and limit psychiatric comorbidities. The only effective methods to eliminate neurofibromas involve physical removal or destruction through surgical resection (ensuring complete removal of the dermal moiety), laser ablation with CO_2_, Erb:YAG or Nd:YAG laser photocoagulation, and electrodissection [2,71,73,74,78]. Laser ablation (especially Erb:YAG) is reported as an effective method of removing large numbers of cutaneous neurofibromas, while maintaining satisfactory cosmetic results [1,2,44,49]. Plexiform neurofibromas are often more difficult to resect due to intimate intertwining with neurovasculature, and there is a high incidence of recurrence [1]. Kim et al. reported the removal of cutaneous neurofibromas in patients with NFt1 using RF with good haemostatic and cosmetic effects, and fast postoperative recovery [71]. The RF, compared to the electrodissection, caused less carbonisation of tissues around the treated skin lesion (smaller necrosis zone). Peltonem et al., based on a multicentre retrospective study, evaluated patients’ global satisfaction of the cutaneous neurofibromas removal with CO_2_ laser, treatment indications, and reasons for withdrawal from the program [78]. The most important indications for removal of the lesions were aesthetic, and pain and itch caused by the tumours. In general, the procedure was well tolerated, and the degree of satisfaction was 8–10 on a scale from 0 to 10. About 30% of patients discontinued the treatment due to organisational constraints, a non-satisfactory aesthetic result, too many lesions to treat, or problems with healing.

Malhotra et al. reported a reduction in size and pain caused by neurofibroma of the hand after topical treatment with sirolimus [76]. Wataya-Kaneda et al. performed a randomised, placebo-controlled study of 18 patients with NF1 using topical sirolimus 0.2% and 0.4% gel. At 24 weeks of treatment, the computed tomography results indicated a dose-dependent tumour volume reduction—the volume reduced in the 0.4% sirolimus group, and there was slower growth in the 0.2% sirolimus group. No significant adverse events were observed, and the major adverse drug reaction was pruritus [77].

#### 3.2.3. Brooke–Spiegler Syndrome (BSS)—Cylindroma, Spiradenoma, Trichoepithelioma, Trichoblastomas

BSS can present with multiple benign adnexal tumours commonly situated on the scalp, face, and neck area, measuring about 0.5 to 3 cm in diameter, and varying from a few to several hundred nodules [1,2]. Therapy options include surgical excision, electrocautery, laser ablation, dermabrasion, and RF [1,2]. Recurrence of the nodules is common, and excessive scarring can occur [94,95,96,97,98].

Both the Erb:YAG and CO_2_ lasers were applied in the removal of trichoepitheliomas in BSS. Some authors prefer Erb:YAG lasers over CO_2_ lasers, due to successful results with ablation, contour, and blend with surrounding tissue [94]. The Erb:YAG laser facilitates the visualisation of hamartomatous tissue while ablating, due to a lack of tissue carbonisation normally seen with CO_2_ lasers. The Erb:YAG laser is more precise, and does not affect the surrounding tissues like the CO_2_ laser does. Thermal damage is minimised during the ablation process, leading to a reduction in scarring.

Botsali et al. reported combination therapy of trichoepitheliomas with the use of ablative laser and imiquimod in patients with BSS with excellent results, especially in terms of lack of recurrence within 1.5 years of follow-up [65]. Urquhart et al. observed the efficacy of 5% imiquimod enhanced by introducing tretinoin after 6 months of treatment in a case of MFT [99]. Alessi et al.’s retrospective study reported a partial response after 32 weeks of 5% imiquimod treatment in two BSS patients [100].

In a case study performed by LoPiccolo et al., the efficacy of various treatments (12 weeks 5% imiquimod; PDT; Erb:YAG; Erb:YAG + 5% imiquimod; Erb:YAG + PDT) was evaluated in single-subject tests on different skin areas [112]. The topical imiquimod in monotherapy was ineffective. Application of Erb:YAG alone or combined with imiquimod brought similar results. Botsali et al. underlined that the discrepancy in those results was caused by a lack of laser-assisted delivery (LAD) approach, as the imiquimod was not applied immediately, instead being applied 2 days after ablation [65]. They further emphasised that the lack of recurrence on long-term follow-up is extremely rare for BSS, and the detected results are remarkable [65]. As imiquimod requires extended treatment durations for trichoepitheliomas, LAD can assist in decreasing the duration.

Tu et al. compared the efficacy of topical sirolimus 1% cream in 30-week monotherapy versus in combination with CO_2_ laser for 52 weeks in two patients with multiple familial trichoepitheliomas. The authors found, respectively, a limitation of disease progression and the limitation of regrowth of trichoepitheliomas [113].

Penev et al. underlined that some of the typical trichoblastoma (TB) lesions may tend to progressively transform into malignant trichoblastic carcinoma, with a potential to metastasise and with aggressive patterns of local growth [129]. Therefore, the authors recommend that complete surgical excision is the first choice treatment. In the case of incompletely excised lesions, local recurrence occurs easily. Mohs micrographic surgery for advanced lesions could also be considered, as the results are usually more satisfactory aesthetically. TB are also excised for cosmetic reasons, or if it occurs in functionally sensitive areas and inflicts functional disorders. Abd Rahim et al. reported laser ablative treatment in patients with multiple TB on the face [95]. Nonsurgical destructive therapies have also been used for multiple small tumours, depending on the lesion sizes, location, and depth. Given the risk of recurrence, patients should continue their follow-up.

It should be also highlighted that both trichoepithelioma and trichoblastoma might imitate BCC in the histopathological picture, as was our patient’s case. Therefore, immunohistochemical analysis and adequate tissue sampling are essential before treatment of suspicious lesions [98]. There is also a publication describing BCC arising on cylindroma in patients with BSS and basaloid squamous cell carcinoma [130,131,132].

According to Table 4, in terms of cylindromas and spiradenomas, various treatment modalities were successfully implemented [1,2,102,103,107]. Removal by conventional surgery with “Scalp-sparing” strategies include early primary excision with direct skin closure, tumour enucleation followed by direct skin closure, and excision followed by secondary intention healing techniques [107,109,110,111]. The wide local excision to avoid the risk of malignant transformation frequently leads to cosmetically unacceptable results, or is impossible to conduct, due to the close distance between numerous tumours. The cryotherapy or deep electrodesiccation of tumours will result in long treatment, depigmentation, and scarring, and these are not acceptable on exposed sites [101]. In electrodesiccation, one can perform only superficial diathermy, which is not very effective even after multiple sittings. Also, laser ablation should be considered for the removal of small tumours. Localised radiotherapy on exposed sites may cause radiation dermatitis, resulting in decreased patient compliance. Chaudhary et al. used RF ablation in the treatment of both cylindromas and trichoepitheliomas [102]. Lucas-Truyols et al. presented a combined procedure of removal cylindromas, comprising the incision and debulking of the tumour, curettage of the remaining tissue of the lesion, electrocautery for haemostasis, and healing by secondary intention, with good cosmetic effect after 12 months [103]. One publication reported an unsuccessful attempt to treat single cylindromas in BSS with topically applied salicylic acid at varying concentrations [104]. Bonadies et al. reported an interesting application of bleomycin electrochemotherapy (ECT) in the treatment of dermal cylindroma in patients with BSS [105]. The ECT provided tumour regression, and improved the patient’s quality of life. The treatment was well tolerated (no pulmonary toxicity revealed), and scalp skin condition significantly improved, regaining a fair follicular density on the margins. After 2 months, an objective response was observed in all the treated areas, with complete response achieved in 25% of the treated lesions and partial tumour regression (30% reduction in tumour size according to the RECIST criteria) in the remaining lesions. The second session was performed after 8 months on the previously treated partially responsive nodules, and new lesions developed outside the treated area—on the scalp, face, and trunk. Moderate facial oedema, pain, skin ulceration, and alopecia were revealed after each session. The late adverse event was the hyperpigmentation on the face and trunk. It is worth mentioning here that the range of ECT applications is divided into the treatment of cutaneous and subcutaneous metastases located in the head and neck of melanoma and NMSC, trunk in case of breast cancer, or non-cutaneous metastases located in bone, liver, or soft tissue sarcoma [133].

#### 3.2.4. Cowden Syndrome (CS)—Trichilemmoma

Due to the benign nature of cutaneous lesions, available treatment options include CO_2_ laser ablation, surgical excision, or treatment with topical 5-fluorouracil [1,2,3,65,66,67,68,69,70]. Alternative therapies comprise electrocautery, cryosurgery, dermabrasion, topic retinoids, interferon 2 alpha, and bleomycin chemotherapy [1,2,3,65,66,67,68,69,70]. Only 50% of patients with CS have facial lesions with features typical of trichilemmomas. Chang et al. proposed the usage of a CO_2_ laser with a pinhole method for patients diagnosed with CS [66]. The pinhole method is based on creating tiny holes penetrating from the epidermis to the deeper dermis with a CO_2_ laser. The authors claim that this method additionally induces regeneration and realignment of collagen bundles, improving the dermal thickness and skin surface texture. Compared with ablative methods, which is associated with shorter procedure and recovery times, there is less post-treatment bleeding and oozing. There are also two reports of successful treatment of oral papillomatosis in CS with a CO_2_ laser and a combination of a Erb:YAG laser with topical sirolimus 0.5% (applied once daily since the fourth course of ablative laser treatment for nine months) to prevent recurrence [67,68].

#### 3.2.5. Phakomatosis Pigmentokeratotica–Nevus Sebaceus Syndrome, Syringocystadenoma Papilliferum, Trichoblastoma

##### Syringocytadenoma Papilliferum (SCAP)

Syringocytadenoma papilliferum (SCAP) is a rare, hamartomatous tumour that arises from the sebaceous glands, with an underlying risk of malignant transformation [4,134,135]. This typically results in BCC, and sometimes squamous cell carcinoma (SCC). There are three different classifications of SCAP: solitary nodular, linear, and plaque. SCAPs present as single or multiple nodular plaque lesions, 40% of which are reportedly associated with a subcutaneous nevus. Approximately 50% occur at birth, up to 30% in adolescence, and the remainder in adulthood [89,134,135]. Syringocystadenocarcinoma papilliferum is a rare malignant differentiation of SCAP with metastatic potential [4]. It should be suspected in lesions with ulcerations and rapid progression. Although successful management of SCAP has been reported with Mohs surgery and CO_2_ laser treatment, surgical excision is ultimately advised due to the risk of malignant transformation. Jordan et al. reported successful treatment of newborns with SCAP of the right ear and neck, treated with two sessions of CO_2_ laser [89].

##### Nevus Sebaceous Syndrome (SN)/Verrucous Epidermal Nevus (VEN)

Contrary to the abovementioned benign skin tumours, the removal of sebaceous or epidermal nevi with different lasers is the best studied in the literature. Many authors reported good results with the use of CO_2_ lasers [136,137,138,139,140,141]. Verma et al. showed 26% of hyperpigmentation and 13% mild atrophic scarring after CO_2_ laser treatment of SN [142]. The complete removal of the facial SN with the fractional CO_2_ laser may include the following pulsed dye laser (595 nm) treatment to prevent scarring after the ablation of the large-sized lesion [79]. Alkhalifah et al. performed a bicentric, retrospective cohort study, including patients (23 VEN, 16 SN) treated with ablative laser with more than a 1-year follow-up [80]. After follow-up ranging between 12 and 127 months, better results, fewer recurrences, and higher patient satisfaction were noted in treatments for VEN than for SN, which had a strong tendency to recur and to develop a scar when treating deeply. Osman et al. performed a comparative randomised clinical study comparing the results of single-session treatment with a CO_2_ laser versus a Erb:YAG laser in in twenty patients with VEN [81]. In the results, both lasers induced noticeable clinical improvement, with no significant differences between the two lasers in treatment response, patient satisfaction, duration of erythema, and side effects. The average time to re-epithelialisation was 13.5 days with the CO_2_ laser and 7.9 days with the Erb:YAG laser (*p* < 0.0005). No scarring was observed in the Erb:YAG laser group and no lesional recurrence was detected in the CO_2_ laser group since treatment.

Levi et al. have indicated excellent results with use of the picosecond 532 nm Nd:YAG laser in the management of six patients with VEN [82]. In one of the treated patients, the authors observed haemorrhagic blisters 2 days after the session. None of the patients experienced recurrence in a 12-month follow-up period, as most recurrences are expected within 3–6 months. Recently, Ponomarev et al. reported successful treatment of SN with dual-length copper vapor laser (CVL) [79]. The authors are convinced that CVL 511-nm (green) radiation eradicates hamartous sebocytes and acanthotic epidermal cells. CVL 578 nm (yellow) radiation inhibits VEGF and provides reliable obliteration of adjacent microvessels, and the treatment provides fast healing without remote scarring of the large-sized, advanced SN on the neck.

Treatment is challenging, and the aim is for optimally clearing the lesion without creating substantial scarring [80]. Surgical removal may prove beneficial for small lesions, yet for large VEN, the significant scarring may provide an inferior cosmetic result. Topical modalities, including corticosteroids, retinoids, calcipotriol, antiproliferative agents (e.g., 5-fluorouracil cream), keratolytics, and chemical peels, as well as intra-lesional corticosteroids, have shown inconsistent efficacy and a high rate of recurrence [83,84,85]. Destructive methods such as lasers, electrodesiccation, and dermabrasion have provided variable efficacy and a high recurrence rate [81,143,144,145]. Cryotherapy alone [146] or combined with CO_2_ lasers has proven beneficial, yet large VEN requires many repetitive treatments and are much less responsive [147].

It is important to underline that during the verrucous stage of sebaceous nevi, a variety of appendageal tumours, like TB, SCAP, or BCC, start to appear, comprising 1% of malignant and 13.6% of benign ones. As a patient ages, tumours progress more quickly and occur more frequently [2].

#### 3.2.6. Muir–Torre Syndrome (MTS)—Sebaceoma, Sebaceous Adenoma, Sebaceous Hyperplasia

The search of MTS and the treatment modalities revealed a single publication regarding sebaceous hyperplasia, which was out of the scope of this review [148].

Oral isotretinoin is effective, but the treatment must continue for several months, and there are high relapse rates with discontinuation. A similar observation was made by Marcusson et al. in a patient diagnosed with MTS, with response restricted to sebaceous hyperplasia [149]. Treatment of sebaceous adenoma with Argon, CO_2_, or KTP lasers are published as case reports out of the scope of MTS [116,117,118,119,120]. Regarding genodermatoses, a single publication regarding treatment of sebaceous adenoma in tuberous sclerosis with use of a CO_2_ laser was reported by Weston et al. [121]. El-Musa et al. reported treatment of extensive and severe sebaceous adenomas with mechanical dermabrasion [122].

#### 3.2.7. Plasma Device Utility in Treatment of Benign Skin Tumours

Based on the research we performed, we were unable to find a single publication reporting the use of a “plasma device” to remove benign skin tumours in presented genodermatoses. Therefore, our case series describing the utility of Plasma IQ^®^ (Berger & Kraft Medical, Ltd., Warsaw, Poland) in the ablation of fibrofolliculomas in BHDS, neurofibromas in NFt1, trichilemmomas in CS, and syringocystadenomas papilliferum in PPK, are to our knowledge, the first published communication, indicating this novel treatment modality in this setting of patients. Based on our good results we have presented another group of skin tumours potentially eligible for this treatment—the sebaceus adenomas and sebaceous hyperplasia in MTS, and in BSS cylindromas, spiradenomas, trichoepitheliomas, and trichoblastomas, though there is a known high risk rate of recurrence in the two letter syndromes.

Plasma IQ (Berger & Kraft Medical, Ltd., Warsaw, Poland) is a modern dermosurgical device certified by the FDA (FDA-cleared) for the removal and coagulation of skin lesions [150]. This device uses an electric arc to sublimate treated tissues. The device is precise and operates only at the site of direct, close contact with the tissue. During the treatment, plasma beams are precisely delivered to the skin using thin needle electrodes, generating microthermal zones that lead to the sublimation of tissues in the treatment area. The recovery period and tissue regeneration after the procedure range from 5 to a maximum of 7 days. Plasma IQ ensures very high precision and, therefore, makes it possible to remove lesions even from the eyelids, avoid extensive scarring and possible complications like deformations. The advantage of plasma over common electrodissection/electrocautery is the smaller depth penetration and increased sublimation diameter. In this study, the damage depth was 193.82 microns, which is less than half of the average epidermis thickness [150]. Moreover, the demarcation of the sublimation site from the surrounding structures is clear and without thermal damage outside this area. All of those features contribute to faster healing and minimising the risk of scarring, which is crucial while treating face surface skin lesions.

If we include the RF devices in the plasma ablation methods, we will have to mention a few publications regarding the aspect of genodermatoses—NFt1, BSS, and Gardner’s syndrome [71,102,103,114,151].

Di Brizzi et al. presented three cases, all out of the scope of this article, describing utility plasma radiofrequency devices in treating seborrheic keratosis, dermal nevus, and acrochordon [152].

## 4. Summary

Patients diagnosed with rare cancer-associated genodermatoses present numerous benign skin tumours, and require periodic supervision due to the increased risk of their malignant transformation, and the propensity to develop solid organ cancers. Unfortunately, guidelines have been developed only for selected genodermatoses, which specify screening and the course of monitoring, along with the frequency of performing individual procedures in different age groups.

Diagnostics requires clinical experience from dermatologists and pathologists, enhanced by the application of appropriate diagnostic methods. The clinical picture is often uncharacteristic, especially among tumours originating from skin appendages. Dermoscopy can provide some support, with differentiation of malignant transformed tumours, or secondary cutaneous neoplasms (BCC, SCC, melanoma). The rarity of genodermatoses and related tumours, accompanied by histopathological overlap of adnexal tumours structures and BCC, results in the need to verify previous diagnoses in some cases.

Both genodermatoses and the treatment of benign skin tumours, present therapeutic challenges for dermatologists and surgeons. In recent years, we have observed a dynamic development of dermatosurgery techniques. The possibility to use the latest laser technologies and other energy sources revolutionised the field of dermatology, offering a versatile and effective tool for the treatment of a wide range of skin conditions such as non-malignant skin tumours. Those technologies not only enhance treatment efficacy but also reduce recovery time, making it a preferred choice for both patients and dermatologists. Unfortunately, concerning the aspect of discussed genodermatoses and associated benign skin tumours, the existing literature predominantly lacks comprehensive data on treatment. It comprises case reports, case series, and single observational studies, which have a lower impact on scientific evidence.

## 5. Conclusions

Healthcare providers need to recognise rare genodermatoses with associated cancers, which are unrevealed and might threaten patients, and their close relatives’ lives. The use of modern dermato-surgical technologies including, presented for the first time in literature, the application of Plasma IQ, makes it possible to improve the aesthetics of rare skin conditions, and, thus, improve the patients’ quality of life.

## 6. Limitations

While this review aims to provide a comprehensive overview of the current literature on selected genodermatoses and the treatment modalities, it is essential to acknowledge certain limitations that may impact our conclusions. The number of available studies varies across different aspects of presented benign adnexal skin tumours, and dermato-surgical treatment including the energy-based devices. The genetic syndromes are a very rare entity; therefore, the overall number and heterogeneity of the available literature could affect the comparability of outcomes between the types of interventions, including the number of treated patients and follow-up period. There is a paucity of comprehensive, large-scale studies dedicated to investigated aspects. The existing literature predominantly comprises case reports, case series, and single observational studies, which have a lower amount of scientific evidence and often lack of comprehensive data on treatment. Collaborative efforts among multidisciplinary teams are vital for formulating evidence-based guidelines.

## Figures and Tables

**Table 1:** Diagnostic criteria of the selected genetic syndromes.

Birt–Hogg–Dubé syndrome (BHDS) [1,2,5]	Major criteria At least five fibrofolliculomas or trichodiscomas, at least one histologically confirmed, of adult-onset Pathogenic *FLCN* germline mutation	Minor criteria Multiple lung cysts: bilateral basally located lung cysts with no other apparent cause, with or without spontaneous primary pneumothorax Renal cancer: early onset (age <50 years) or multifocal or bilateral renal cancer, or renal cancer of mixed chromophobe and oncolytic histology A first-degree relative with BHD	A diagnosis of BHG is established with either one major criteria or two minor criteria
Brooke–Spiegler syndrome (BSS) [1,2,5,6]	There is a lack of standardised diagnostic criteria		Diagnosis necessitates consideration of family history, clinical examination, histological findings, and genetic analysis
Cowden syndrome (CS) [1,2,5,7]	Major criteria Breast cancer Endometrial cancer Follicular carcinoma of the thyroid gland Gastrointestinal hamartomas (≥3, including ganglioneuromas, but excluding hyperplastic polyps) Adult-onset Lhermitte–Duclos disease Macrocephaly (>97th percentile: 58 cm in women and 60 cm in men) Macular pigmentation of the glans penis Multiple mucocutaneous lesions (any of the following): trichilemmomas (≥3, at least one biopsy proven) acral keratoses (≥3, palmoplantar keratotic pits and/or acral hyperkeratotic papules) mucocutaneous neuroma (≥3) oral papillomas (≥3, particularly on gingiva and tongue)	Minor criteria Autism spectrum disorder Colorectal cancer Oesophageal glycogenic acanthosis (≥3) Lipoma (≥3) Intellectual disability (IQ ≤ 75) Renal cell carcinoma Testicular lipomatosis Thyroid cancer (papillary carcinoma or follicular variant of papillary) Thyroid structural lesions (adenoma, adenomatous goitre, etc.) Vascular anomalies (e.g., multiple developmental venous anomalies)	A diagnosis of CS is established if the patient fulfils one of the following criteria: Three or more major criteria, one of which is macrocephaly, adult-onset Lhermitte–Duclos disease, or gastrointestinal malrotation Two or more major criteria and three or more minor criteria
Muir–Torre syndrome (MTS) [1,2,3,5,8]	The diagnosis is made upon the detection of at least one sebaceous tumour and one internal malignancy during any period of the patient’s life while ensuring that other factors such as radiotherapy or immunosuppression are ruled out.		Diagnosis may also be supported by the presence of multiple keratoacanthomas, a known visceral malignancy, and family history indicative of MTS
Neurofibromatosis type 1 (NFt1) [1,2,5,9]	A: The diagnostic criteria are met in an individual who does not have a parent diagnosed with NF1 if two or more of the following are present: Six or more café-au-lait macules over 5 mm in greatest diameter in prepubertal individuals and over 15 mm in greatest diameter in post-pubertal individuals Freckling in the axillary or inguinal region Two or more neurofibromas of any type or one plexiform neurofibroma Optic pathway glioma Two or more iris Lisch nodules identified by slit lamp examination or two or more choroidal abnormalities—defined as bright, patchy nodules imaged by optical coherence tomography (OCT)/near-infrared reflectance (NIR) imaging A distinctive osseous lesion such as sphenoid dysplasia, anterolateral bowing of the tibia, or pseudarthrosis of a long bone A heterozygous pathogenic NF1 variant with a variant allele fraction of 50% in apparently normal tissue such as white blood cells	B: A child of a parent who meets the diagnostic criteria specified in A merits a diagnosis of NF1 if one or more of the criteria in A are present.	
Phacomatosis pigmentokeratotica (PPK) [1,2,5,10]	Skin: Organoid epidermal nevus (sebaceous differentiation) Speckled lentiginous nevus (i.e., a café-au-lait macule superimposed by multiple dark papules representing melanocytic nevi)	Extra-cutaneous anomalies including ocular, musculoskeletal, and nervous system abnormalities	

**Table 2:** Genetic and clinical characteristics of selected genodermatoses in the context of cutaneous and extra-cutaneous malignancies.

Disorder	Gene Symbol	Gene or Gene Product	Mode of Inheritance	Benign/Malignant Skin Tumours	Spectrum of Extra-Cutaneous Neoplasm	References
BHDS	FLCN	Folliculin	AD	Fibrofolliculoma, trichodiscoma, acrochordon	Renal cancer, melanoma, mesothelioma	[1,2,5,7,11,12]
CS	PTEN	Phosphatase and tensin homolog	AD	Trichilemmoma, oral papillomatosis, neuroma, acral keratosis, macular pigmentation of the glans penis, lipoma	Breast, thyroid, endometrial, colon, kidney cancer, melanoma, hamartoma-type lesions in various internal organs (breast, thyroid, uterus, gastrointestinal tract, brain, mucous membranes)	[1,2,5,12,13,14]
PPK	HRAS	HRAS/p21s: V-HA-RAS Harvey rat sarcoma p21s GTPase		Nevus sebaceous, speckled lentiginous nevus, basal cell carcinoma, melanoma	Malignancies in individuals with PPK display urologic and nephrological tropism	[1,2,5,15]
MTS	MSH2, MLH1, MSH6	MutS homolog 2 (mismatch repair enzyme) MutL homolog 1 (mismatch repair enzyme) MutS homolog 6 (mismatch repair enzyme)	AD	Sebaceoma, sebaceous adenoma, sebaceous carcinomas, keratoacanthoma, basal cell carcinoma	Colon carcinoma	[1,2,3,5,8,16]
NFt 1	RAS, FGFR3, PIK3CA	Neurofibromin 1 (GTPase activating protein)	AD	Café-au-lait, cutaneous, subcutaneous, plexiform neurofibromas, juvenile xanthogranulomas “freckling” in the axillary and groin regions (Crowe’s sign)		[1,2,5,17,18]
BSS	CYLD	CYLD (deubiquitinating enzyme)	AD	Cylindromas, spiradenomas, trichoepithelioma basal cell carcinoma		[1,2,5,6]

**Table 3:** The origin of selected benign skin tumours of the spectrum of presented genodermatoses, with immunohistochemistry differentiation, and dermoscopic structures, based on, respectively, Ho and Collie 2023 [25], and published data.

Tumour	Origin/IHC Staining	Dermoscopy	References
Cylindroma	Hair bulge PAS(+), SOX10(+), MYB(+), CD117(+)	Unfocused arborising vessels, blue–grey dots/ globules, faint greyish–bluish hue, whitish areas	[23,24,26,27]
Fibrofolliculoma/Trichodiscoma	Mantle of the hair follicle fibrocytes CD34(+), S100(−)	FF—well-demarcated whitish areas/globules of pallor with central yellowish-brown spot (follicular opening), curvilinear vessels connecting red dots and globules TD—white globular structures, blue–grey nests, blurred linear vessels	[24,28,29]
Neurofibroma	Schwann cells S100(+), SOX10(+) fibroblasts CD34(+)	Pink–red structureless areas, scar-like areas, a peripheral halo of brown homogeneous pigmentation, fingerprint-like structures, fissures	[30,31]
Nevus sebaceous syndrome	Hamartoma with epidermal, sebaceous, and adnexal gland differentiation HRAS(+)	Yellowish-brown globules, fissures and ridges arranged in “cerebriform pattern”, comedo-like openings, and milia-like cysts	[24,32]
Sebaceous adenoma	Sebocytes of sebaceous gland cells with less than 50% germinative cells component Adipophilin(+), EMA(+)	Central opaque whitish structure, yellowish, peripheral telangiectasias (crown vessels), arborising vessels with whitish structures resembling cotton flaks and yellow–orange background	[23,24,33]
Sebaceoma	Sebocytes of sebaceous gland cells with over 50% germinative cells component Adipophilin(+), EMA(+), Ber-EP4(-)	Arborising vessels with whitish structures resembling cotton flaks and yellow–orange background	[24,33,34]
Spiradenoma	Hair bulge Luminal cells SOX10(+), c-KIT(+), EMA(+), CEA(+), cytokeratins(+); myoepithelial cells calponin(+), SMA(+), p63(+)	Homogeneous pink or pink–orange area with arborising telangiectasias, blue ovoid nests or blue globules	[24,26,27]
Syringocystadenoma papilliferum	Eccrine gland cells BRAF V600E(+), luminal cells CEA(+), EMA(+), outer cell layer calponin(+), SMA(+), p63(+)	Polymorphous vascular pattern, perivascular whitish halos, pinkish white backgrounds, symmetric erythematous lesion with exophytic papillary structures, ulceration or central depression, corresponding to cystic spaces open to skin surface on histopathology (hp)	[23,24]
Trichilemmoma	Hair follicle outer root sheath cells PTEN(+), p16(+), CD34(+)	Keratin masses, perivascular whitish halos, radial linear vessels, or hairpin ones, with distal thickening adopting a triangular form in the periphery (red iris-like structure), white shiny areas surrounding those vessels, central hyperkeratotic mass/crust/depression	[23,24,35]
Trichoblastoma	Follicle germ cell Ber-EP4(+), PHLDA1(+)	Arborising vessels and blue–grey dots or globules or ovoid nests (corresponding to pigmented basaloid nodules on hp)—BCC imitator, ivory white background though entire lesion (corresponding to desmoplastic stroma on hp) with arborising vessels, keratin cyst or shiny white streaks, in adamatoid variant of TB (previously named cutaneous lymphadenoma) pink papule with arborising vessels on orange background and peripheral dotted and glomerular vessels	[23,24,36,37,38]
Trichoepitelioma	Follicle germ cell with smaller aggregations of cells Ber-EP4(+), PHLDA1(+)	Arborising vessels, shiny white areas, thin in focus arborising vessels, shiny white areas/background, milia-like cysts (corresponding to keratin cysts on hp), sporadically also rosettes, blue–grey dots and yellowish-brown background	[23,24,36,38,39]

**Table 4:** Summary of treatment modalities of benign skin tumours, including setting of selected genodermatoses.

Tumour Type	Dermatosurgical Treatment	Topical Treatment	References
Fibrofolliculoma	Dermabrasion, laser ablation/resurfacing (Erb:YAG, CO_2_, fractional CO_2_, copper vapor laser)	Sirolimus 0.1%	[55,56,57,58,59,60,61,62,63,64]
Trichilemmoma	Surgical excision, dermabrasion, laser ablation (CO_2_), curettage, electrocautery, Mohs micrographic surgery	Sirolimus, 5-fluorouracil, laser-assisted delivery of imiquimod, retinoids	[3,65,66,67,68,69,70]
Neurofibroma	Surgical excision, laser ablation (CO_2_, Erb:YAG, Nd:YAG), RF ablation, electrodissection	Sirolimus 0.2%, 0.4%	[2,71,72,73,74,75,76,77,78]
Epidermal nevus/nevus sebaceous syndrome	Laser ablation (CO_2_, Erb:YAG, picosecond Nd:YAG, copper vapour), surgical excision	Sirolimus, corticosteroids (topical, intra-lesional), retinoids, calcipotriol, 5-fluorouracil, keratolytics, chemical peels	[72,79,80,81,82,83,84,85,86,87,88]
Syringocystadenoma papilliferum	Surgical excision and laser ablation (CO_2_)	Not reported	[89,90,91,92,93]
Cylindroma	Surgical excision, Mohs micrographic surgery, dermabrasion, cryotherapy, RF ablation, electrocautery	Electrochemotherapy with bleomycin, intralesional triamcinolone acetonide, both topically and systemically applied acetylsalicylic acid, 5% imiquimod	[65,94,95,96,97,98,99,100,101,102,103,104,105,106,107,108,109,110,111]
Trichoepithelioma	Surgical excision, laser ablation (Erb:YAG, CO_2_, KTP 532 nm), electrocautery, cryosurgery, dermabrasion	5% imiquimod ± tretinoin, sirolimus, laser-assisted sirolimus	[65,97,99,100,112,113,114,115]
Spiradenoma	Surgical excision, laser ablation (Erb:YAG, CO_2_, KTP), electrocautery, RF ablation	Laser (fractional Erb:YAG)-assisted delivery of 5% imiquimod	[6,65,72,94,95,96,97,98,99,101,102,103,106,107,108,109,110,111,114]
Sebaceous adenoma	Laser ablation (Argon, CO_2_, KTP), dermabrasion, PDT	Not reported	[116,117,118,119,120,121,122,123]

## Data Availability

Data are unavailable due to privacy.
